# Combination of Two Synchrotron Radiation-Based Techniques and Chemometrics to Study an Enhanced Natural Remineralization of Enamel

**DOI:** 10.1021/acs.analchem.1c05498

**Published:** 2022-03-23

**Authors:** Sandra Diez-García, María-Jesús Sánchez-Martín, José Manuel Amigo, Manuel Valiente

**Affiliations:** † GTS Research Group, Department of Chemistry, Faculty of Science, Universitat Autònoma de Barcelona, 08193 Bellaterra, Spain; ‡ Ikerbasque, Basque Foundation for Science, María Díaz de Haro, 48013 Bilbao, Spain; ∥ Department of Analytical Chemistry, University of the Basque Country UPV/EHU, P.O. Box 644, 15 48080 Bilbao, Basque Country, Spain

## Abstract

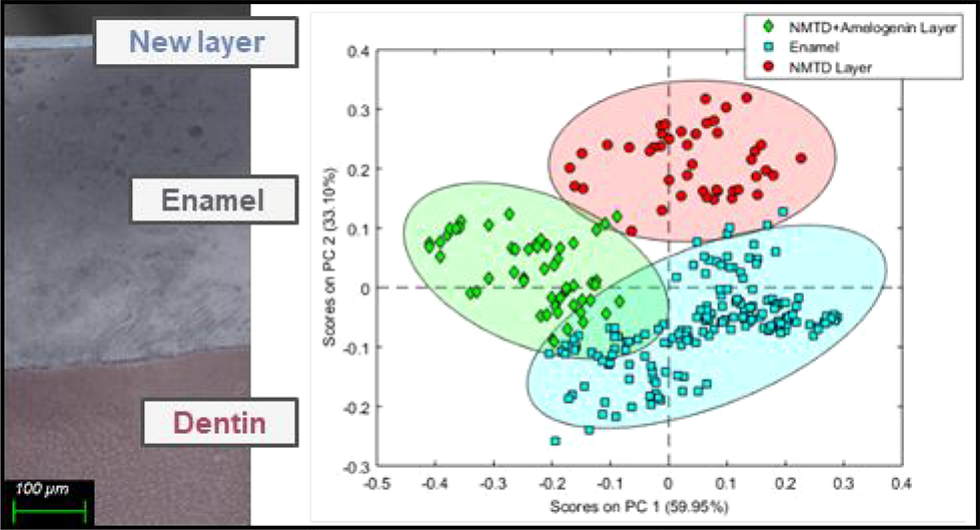

The limitations to assess dental enamel remineralization have been overcome by a methodology resulting from the appropriate combination of synchrotron radiation-based techniques on both, infrared microspectroscopy and micro X-ray diffraction, with the help of specific data mining. Since amelogenin plays a key role in modulating the mineralization of tooth enamel, we propose a controlled ion release for fluorapatite structural ions (Ca^2+^, PO_4_^3–^, and F^–^, also including Zn^2+^) by using weak acid and weak base ion-exchange resins in the presence of amelogenin to remineralize the surface of etched teeth. This combination provides the necessary ions for enamel remineralization and a guide for crystal growth due to the protein. Remineralized tooth samples were analyzed by applying the indicated methodology. The synchrotron data were treated using principal component analysis and multivariate curve resolution to analyze the mineral layer formed in the presence and absence of amelogenin. The remineralizing treatment created a fluorapatite layer free of carbonate impurities and with a similar orientation to that of the natural enamel thanks to amelogenin contribution.

## Introduction

Tooth enamel is the most mineralized and hardest tissue in the human body. The enamel is constituted by multiple rodlike apatite crystals, which are arranged in ordered prisms.^1,2^ Mineral composition of mature enamel is a mixture of compounds, primarily hydroxyapatite (HA), which is crystalline calcium phosphate (Ca_10_(PO_4_)_6_(OH)_2_) that has a hexagonal crystal system with the *P*6_3_/*m* space group.^3,4^ This enamel HA is not stable and suffers changes due to chemical interactions. The major change is produced when phosphate is substituted by carbonate because this substitution increases the solubility of the enamel. However, if the hydroxyl ions of HA are replaced by fluoride ions, fluorapatite (FA), a more acid-resistant compound, is generated.^5^

Several techniques have been used to assess tooth remineralization, such as scanning electron microscopy, atomic force microscopy, or indentation. However, these techniques have certain limitations regarding the analysis of the composition of enamel remineralization and crystal orientation.^6,7^ Therefore, synchrotron radiation-based infrared microspectroscopy and microdiffraction were used for this study to overcome these constraints.

Fourier transform infrared (FTIR) spectroscopy is a well-recognized molecular vibrational technique that has been widely used to investigate the chemical structural properties of natural materials.^8,9^ It has been extensively used for mapping the material properties of mineralized tissues such as dental enamel, including mineralization, crystallinity, or carbonate substitution.^10^ The incorporation of a microscope in FTIR microspectroscopy (μFTIR) has introduced the possibility of combining biochemical and spatial information. Coupling this technique to a synchrotron radiation light source, synchrotron radiation-based FTIR microspectroscopy (SR-μFTIR), allows a much better signal-to-noise ratio and the use of a smaller beam size without losing signal efficiency, since one of the most outstanding properties of a synchrotron radiation source is its high brightness compared with a traditional globar source.^11^

On the other hand, as apatites have a well-defined hexagonal crystal structure, diffraction methods can be used to analyze the crystal structure in teeth.^1^ The crystallographic properties of enamel and fluoridated enamel have been investigated using X-ray diffraction (XRD). Incorporating fluoride into the enamel structure may lead to crystallographic changes in enamel (contraction of the *a*,*b*-axis, increase in apatite crystal size and reduction of crystal defects). Nevertheless, enamel crystallinity varies at different layers. If the enamel is ground into powder mixing them, it may affect the accuracy of the measurement.^12^ The micro X-ray diffraction (μXRD) analysis overcomes the limitations of conventional powder XRD and can obtain site-specific data directly from the tooth allowing the study of the enamel crystallinity at different levels.^13^ The synchrotron through-the-substrate micro X-ray diffraction (tts-μXRD) used in the present work allows performing punctual analysis in thin sections with a spot size of few micrometers. Therefore, this technique enables data collection directly on thin tooth sections preserving the textural context and allowing local identification of mineral phases.^14^

There are several factors that put oral health at risk, such as the popularity of whitening systems with their side effects or the excessive consumption of acidic foods and beverages.^15−18^ Demineralization caused by regular exposure of the tooth enamel to acids, such as those produced within accumulations of bacterial plaque from dietary carbohydrates, removes mineral ions from HA crystals and may cause a caries lesion. Demineralization in early caries lesions can be reversed by calcium and phosphate in saliva.^19−22^ However, mature tooth enamel is acellular and does not regenerate itself after substantial loss.^23,24^ Mechanical properties of the macroscopic enamel tissue are highly dependent on the alignment and orientation of HA crystals. Therefore, changes in surface structure as a result of caries or microscopic damage can cause significant impairment of the dental function and, ultimately, tooth loss.^1^ More than 12 million dental implants are needed annually worldwide as part of routine oral rehabilitation.^25^

Fluoride is the most popular agent for enhancing remineralization. Besides the antibacterial properties at low concentrations, fluoride stops the demineralization and favors the opposite process of remineralization on the tooth surface.^20^ During remineralization processes, fluoride ions promote the formation of FA in the presence of calcium and phosphate ions. At higher concentrations, it creates a calcium fluoride layer that acts as a reservoir source for fluoride and protects the enamel from the formation of caries.^26^ Therefore, fluoride is added to toothpastes, mouthwashes, and drinking water as an anticaries agent.^20^ Despite its benefits, high concentrations of fluoride can cause undesirable side effects and unfortunately, in many dental products, fluoride ions are released too rapidly, producing high concentrations in a brief period due to the short oral application time.^27,28^

This research employs an innovative approach against dental demineralization to avoid the side effects of high fluoride concentrations in the oral environment and extend the contact time between the fluoride ions and the tooth surface, enabling successful remineralization. The product called NMTD^29^ (new material for dental treatment, from its Spanish acronym Nuevo Material de Tratamiento Dental) provides a controlled release system for the anticaries treatment and it is composed of a combination of weak acid and weak base ion-exchange resins loaded with calcium, fluoride, phosphate, and zinc. This agent allows the formation of FA by controlling the rate of fluoride release into the oral environment, in conjunction with the release of calcium and phosphate ions to induce remineralization. The molar ratio of the calcium, fluoride, and phosphate ions has to be close to that of the organomineral tissue to be remineralized. Zinc ions act as initiators of the ionic release of the structural ions. In addition, zinc incorporation into enamel may accelerate its remineralization and reduce the rate of enamel demineralization, and zinc also has antibacterial and malodor control properties.^21,30^

Furthermore, enamel matrix proteins play a vital part during the development of enamel in the regulation of mineralization and crystal organization. Then, the protein matrix is proteolytically degraded during the enamel maturation stage. Amelogenin constitutes more than 90% of the organic matrix, being the most abundant protein in the forming enamel. The importance of the amelogenin protein is well-known because amelogenin self-assembly controls the morphology, size, and orientation of the growing crystals.^23^ For this reason, amelogenin in conjunction with NMTD is used in this research to achieve a remineralization similar to that of natural enamel.

This study aims to evaluate the efficiency of the remineralization after the treatment with NMTD and amelogenin and to determine the protein influence on the morphological changes of the remineralized enamel. Synchrotron infrared microspectroscopy and microdiffraction, joined with the proper chemometric method, allow us to study the evolution of the structure of apatites and their distribution after the remineralization process. The chemometric methods used are principal component analysis (PCA) and multivariate curve resolution (MCR). PCA is a variable reduction tool to identify trends and patterns in an extremely efficient manner by analyzing the variance of the data.^31^ On the contrary, MCR allows us to understand the physical or chemical differences of the mineral formed in the presence and absence of amelogenin.^32^

## Experimental Section

### *In Vitro* Dental Remineralization

All the reagents used are detailed in Supporting Information section 1.1. Bovine teeth are used as a model, given their similarity to human teeth.^33,34^ Bovine tooth specimens were cleaned of gross debris before removing the root with a diamond saw (South Bay Technology, San Clemente, CA). The resulting tooth samples were embedded in a Paladur clear autopolymerizing acrylic resin (Heraeus Kulzer, Hanau, Germany), closing the root aperture and leaving the front surface exposed. The embedded teeth were then etched with hydrochloric acid 1 M for 30 s to mimic the early stage of dental erosion and immediately afterward cleaned by rinsing with Milli-Q water while brushing for 20 s with an electric toothbrush.

Artificial saliva was prepared by mixing the following compounds in the indicated concentrations: potassium chloride 0.24 g/L, calcium chloride dihydrate 0.078 g/L, potassium dihydrogen phosphate 0.544 g/L, magnesium chloride hexahydrate 0.041 g/L, HEPES 4.77 g/L. After complete dissolution of the saliva components, the pH was adjusted to 7.1 ± 0.4 with potassium hydroxide pellets.

In order to perform the remineralizing treatments, artificial saliva was added to NMTD until the consistency of dense gel was reached for its application to bovine teeth. In the case of the treatment with NMTD and amelogenin (synthesis detailed in Supporting Information section 1.3), 100 μg/mL of human amelogenin was added to the saliva before mixing with NMTD. In both cases the mixture of NMTD with the corresponding saliva (with or without amelogenin) was distributed on the enamel surface of acid-etched teeth, and then the samples were placed in a sealed vessel. The base of this container was filled with saliva to maintain the humidity of the environment and the recipient was placed inside an incubator at the normal temperature of the oral cavity (37 °C). Every 24 h for 15 days, each treatment was renewed by washing the samples carefully with Milli-Q water and brushing for 20 s before applying a fresh treatment portion. Finally, all teeth were cleaned by brushing with Milli-Q water for 20 s and stored in a 0.5% chloramine T solution until the preparation for the synchrotron experiments.

### Specular Reflectance SR-μFTIR Experiment

Two samples of each treatment were completely embedded in the same Paladur acrylic resin and longitudinally cut in two halves with a Struers Minitom precision diamond saw (Copenhagen, Denmark) to obtain a mesial view from the inside. A sequence of silicon carbide paper was used to polish the exposed tooth area with a Struers LaboPol-25 polishing machine (Copenhagen, Denmark), starting at grit size P2500 and increasing to P4000, under a constant flow of tap water. A sequence of diamond suspensions with a mean particle size of 3 and 1 μm was used to finish the polishing and obtain the appropriate reflection properties for the measurements. A sample image can be observed in the Supporting Information Figure S1a.

Tooth samples were analyzed by μFTIR coupled to synchrotron radiation (SR-μFTIR) in reflectance mode. The experiment was carried out at MIRAS beamline of ALBA Synchrotron (Cerdanyola del Vallès, Spain)^35^ using a Hyperion 3000 microscope coupled to a Vertex 70 spectrometer (Bruker, Ettlingen, Germany) and equipped with a mercury-cadmium-telluride (MCT) detector. More details of data acquisition and data treatment are shown in Supporting Information section 1.4.

### Synchrotron tts-μXRD Experiment

A couple of samples of each treatment were released from the Paladur acrylic resin and embedded in Epofix resin (Struers, Copenhagen, Denmark). The tooth samples were longitudinally cut in half, fixed on a glass substrate of 1.5 mm thickness, and polished to reduce the sample thickness down to 30 μm and achieve a flat surface. A tooth thin section image can be seen in the Supporting Information Figure S1b. These sections were analyzed by synchrotron tts-μXRD. The experiments were performed at the Materials Science and Powder Diffraction (MSPD) beamline of ALBA Synchrotron (Cerdanyola del Vallès, Spain).^36^ The MSPD beamline is equipped with Kirkpatrick–Baez mirrors and a Rayonix SX165 CCD detector. More details of data acquisition and data treatment are shown in Supporting Information section 1.5.

## Results and Discussion

### Specular Reflectance SR-μFTIR

After 15 days of the remineralizing treatments, teeth had a new mineral layer between 20 and 30 μm of thickness measurable with the microscope of MIRAS beamline. Measurements were taken on a frame including points of the enamel and of this new layer to study the similarities and differences of the crystals of the newly formed layers for the different treatments (NMTD with amelogenin or NMTD alone) with the underlying enamel.

**Figure 1:**
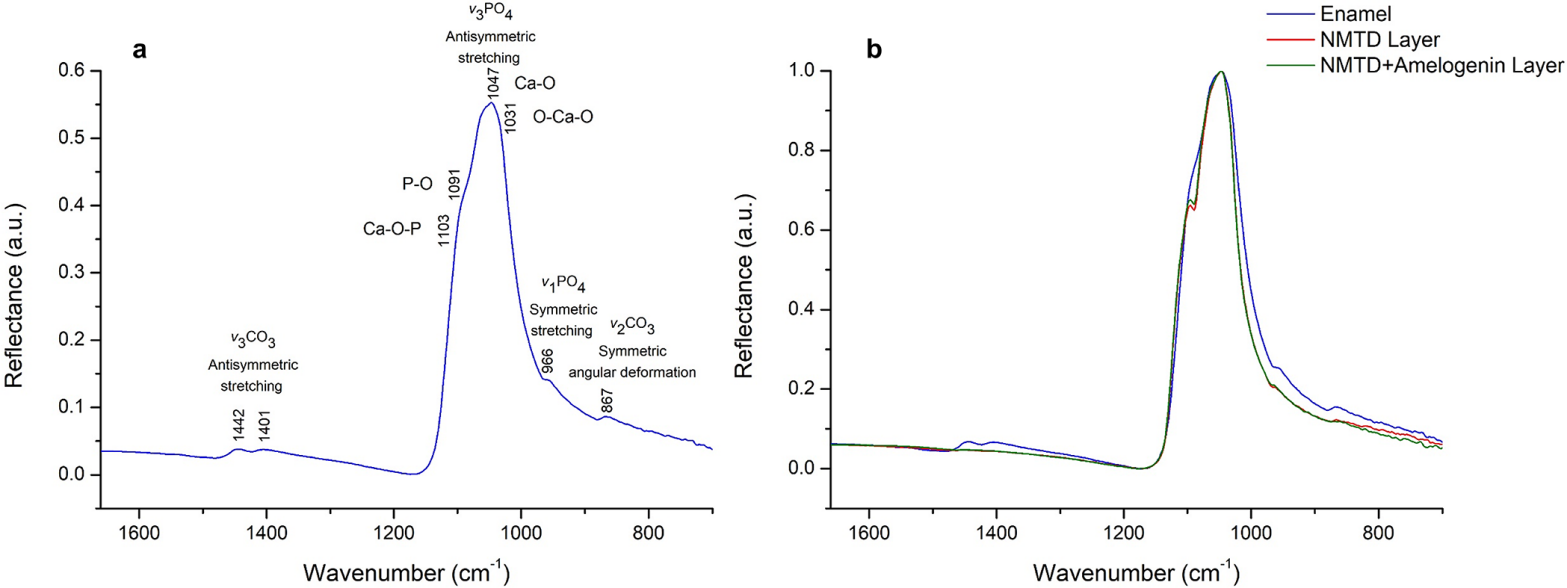
(a) Average of the specular reflectance FTIR spectra of enamel and band assignment numbers (*n* = 11). (b) Average of spectra corresponding to enamel, NMTD layer with amelogenin, and NMTD layer without amelogenin normalized to a maximum (*n* = 11).

Infrared spectroscopy of apatites generally provides two different kinds of information. The crystalline quality is evaluated from the width of the absorption bands due to the phosphate vibrational modes. The other type of information is based on the presence of molecular species, like carbonate groups, which are detected by specific vibrational bands.^37^ Bands corresponding to carbonates and phosphates in the enamel HA are identified in Figure 1a. The bands associated with the ν_3_PO_4_ vibrations (antisymmetric stretching) are observed between 1170 and 965 cm^–1^.^38,39^ In the literature, the deconvolution of the ν_3_PO_4_ bands in stoichiometric apatite is described as secondary phase vibrations of Ca–O–P at 1103 cm^–1^, P–O at 1091 cm^–1^, Ca–O at 1047 cm^–1^, and O–Ca–O at 1031 cm^–1^.^40^ The weak ν_1_PO_4_ band (symmetric stretching) is present at 966 cm^–1^.^37^ The region between 1580 and 1320 cm^–1^ corresponds to ν_3_CO_3_ antisymmetric stretching, where two maximums at 1442 and 1401 cm^–1^ can be appreciated.^8^ An additional small band related to structural carbonates (ν_2_CO_3_ symmetric angular deformation) is present at 867 cm^–1^.^8,37,39^ Carbonate can substitute into two anionic sites of the HA structure. In carbonated apatite type B, which is the predominant type in biological apatite, it is located at phosphate group sites, while in carbonated apatite type A, it is found at hydroxyl group sites.^41^ According to the peak deconvolution from the literature, the ν_3_CO_3_ vibration in carbonated apatite type A splits into two peaks at 1530 and 1465 cm^–1^. In contrast, type B can be characterized by peaks at 1456 and 1423 cm^–1^ in the IR spectrum.^41,42^ In the case of the ν_2_CO_3_ out-of-plane bending mode, a peak at 879 cm^–1^ is reported to belong to type A and another at 872 cm^–1^ to type B.^41,43^ The combined presence of A and B carbonates affects the carbonate peak positions compared to apatite with only A or only B type.^41^ Meaning that the carbonate peaks detected in the regions 1580–1320 cm^–1^ and 880–830 cm^–1^ of the enamel in reflection mode show a mixture of type A and B carbonates.

Both types of layers (Figure 1b) present the characteristic peak of apatites due to the phosphate group near 1050 cm^–1^, indicating a high crystallinity.^44^ In both layers, there is an absence of the two small peaks near 1400 cm^–1^ and the small peak at 867 cm^–1^ that are present in the enamel due to the carbonate group.^45^ The lack of the carbonate substitution group of high solubility and the narrowing of the phosphate peak in both layers indicate conversion to a more highly crystalline apatite compared to enamel. In addition, the layers appear to have a more evident vibration at 1103 cm^–1^ than the enamel, corresponding to the Ca–O–P secondary phase vibration. This could be due to a higher amount of calcium interacting with phosphate than in enamel since there are no carbonates in the layer, and therefore there is no calcium bound to carbonate as in enamel. Further evidence is included in the Supporting Information Figure S2.

**Figure 2:**
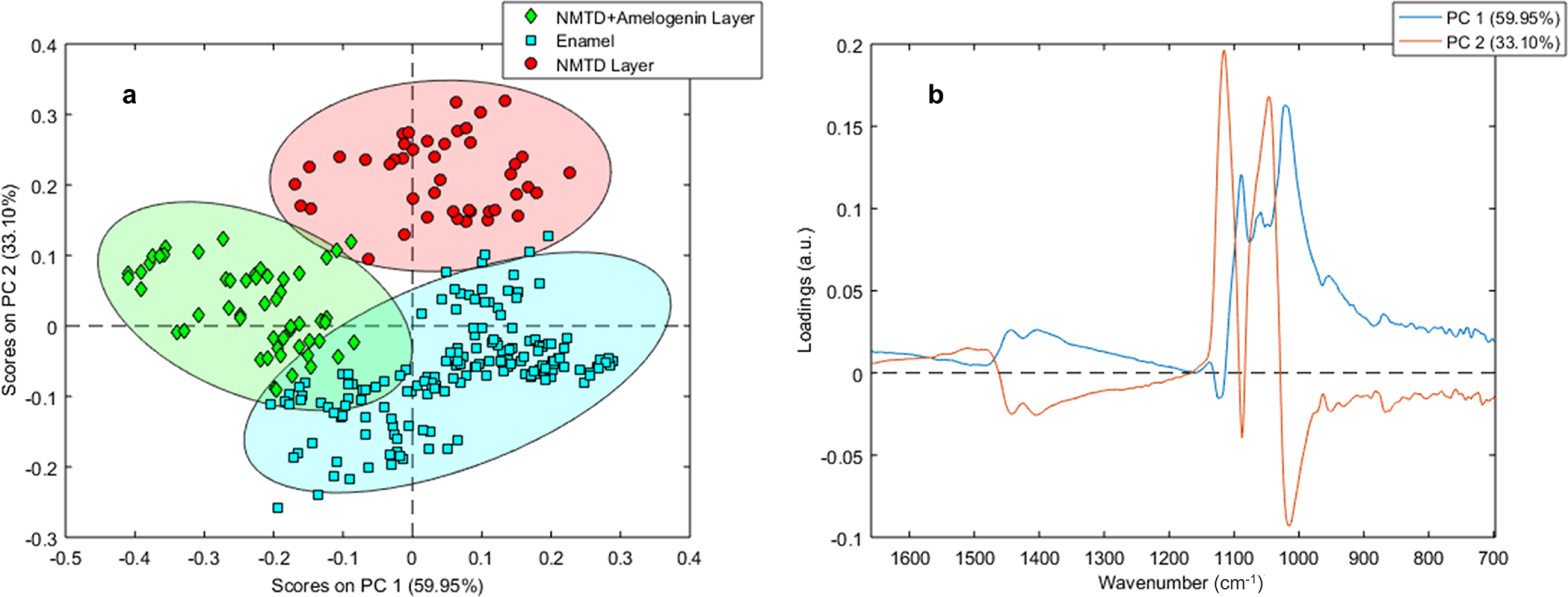
(a) PCA scores graph of the FTIR spectra from the enamel and layer of two samples with amelogenin and two samples without amelogenin. (b) PC 1 and PC 2 loadings of the PCA.

In Figure 2a, the PCA of spectra corresponding to enamel and both layers shows that principal component 1 (PC 1) separates the two treatments (NMTD with and without amelogenin) from each other and principal component 2 (PC 2) separates the enamel from the treatments as it separates according to the depth of the measurement. PC 1 represents 59.95% of the variation, while PC 2 accounts for the 33.10%. In Figure 2b, the loadings for PC1 and PC2 show the main peaks in the regions 1580–1320 cm^–1^ and 1170–965 cm^–1^, corresponding to the ν_3_CO_3_ and the ν_3_PO_4_ vibrations. Therefore, these vibrations are responsible for the scores separation in Figure 2a. The points in the PCA belonging to the layer with protein are statistically closer to the enamel points than those belonging to the layer with NMTD alone, which may be due to the 1103 cm^–1^ shoulder being more pronounced in the NMTD layer (Figure 1b).

### Synchrotron tts-μXRD

**Figure 3:**
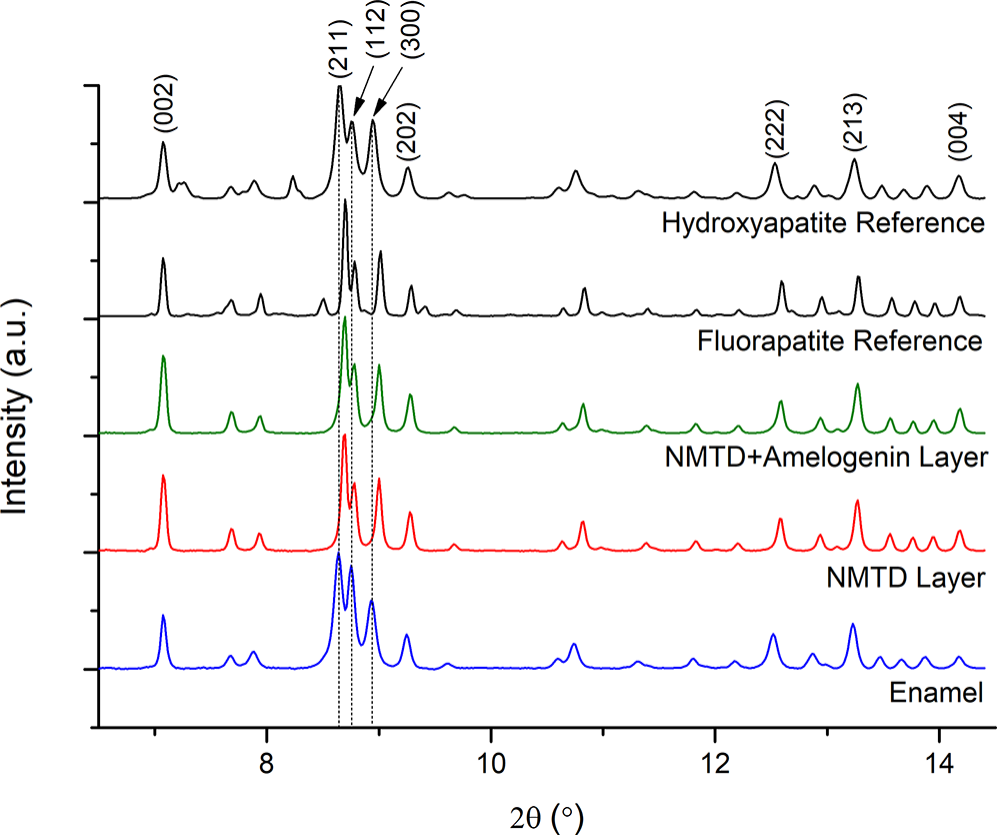
Diffractograms obtained by synchrotron tts-μXRD for HA reference, FA reference, enamel, NMTD layer with amelogenin, and NMTD layer without amelogenin, normalized to maximum and showing reflections.

A typical diffraction pattern of HA was found inside the enamel, the main (*hkl*) Miller indices^4,46^ (002), (211), (112), (300), (202), (222), (213), and (004) are indicated in Figure 3. A shift to a slightly higher angle was observed in the (211), (112), and (300) reflections of the external layer and the FA reference (synthesis explained in Supporting Information section 1.2), compared to the enamel and the HA reference, as expected from the slight reduction in the *a*,*b*-axis dimension when F^–^ ions replaces OH^–^ ions.^47,48^ This shift confirmed the substitution of fluoride ions into the apatite lattice, therefore the remineralized layer formed is composed of FA, more resistant than enamel HA.

Synchrotron tts-μXRD was also used to investigate the texture (or preferred orientation) of enamel crystallites in the tooth sections. The preferred orientation refers to the degree of alignment of the crystallites. The intensities of different XRD peaks can be employed to estimate the macroscopic level-specific orientation of crystals. Sharp and intense (002) and (004) peaks in the apatite, as can be seen for the new layers in Figure 3, indicate that crystals prefer to be aligned along the *c*-crystallographic axis as in real enamel.^49,50^ The lattice planes (002) and (004) along the *c*-axis of the enamel crystals are oriented perpendicular to the tooth surface, following the direction of the enamel prism arrangement.^1,51^ Perpendicularity to the enamel surface maximizes the strength and bending capability and enhances the wear resistance capacity.^52^

**Figure 4:**
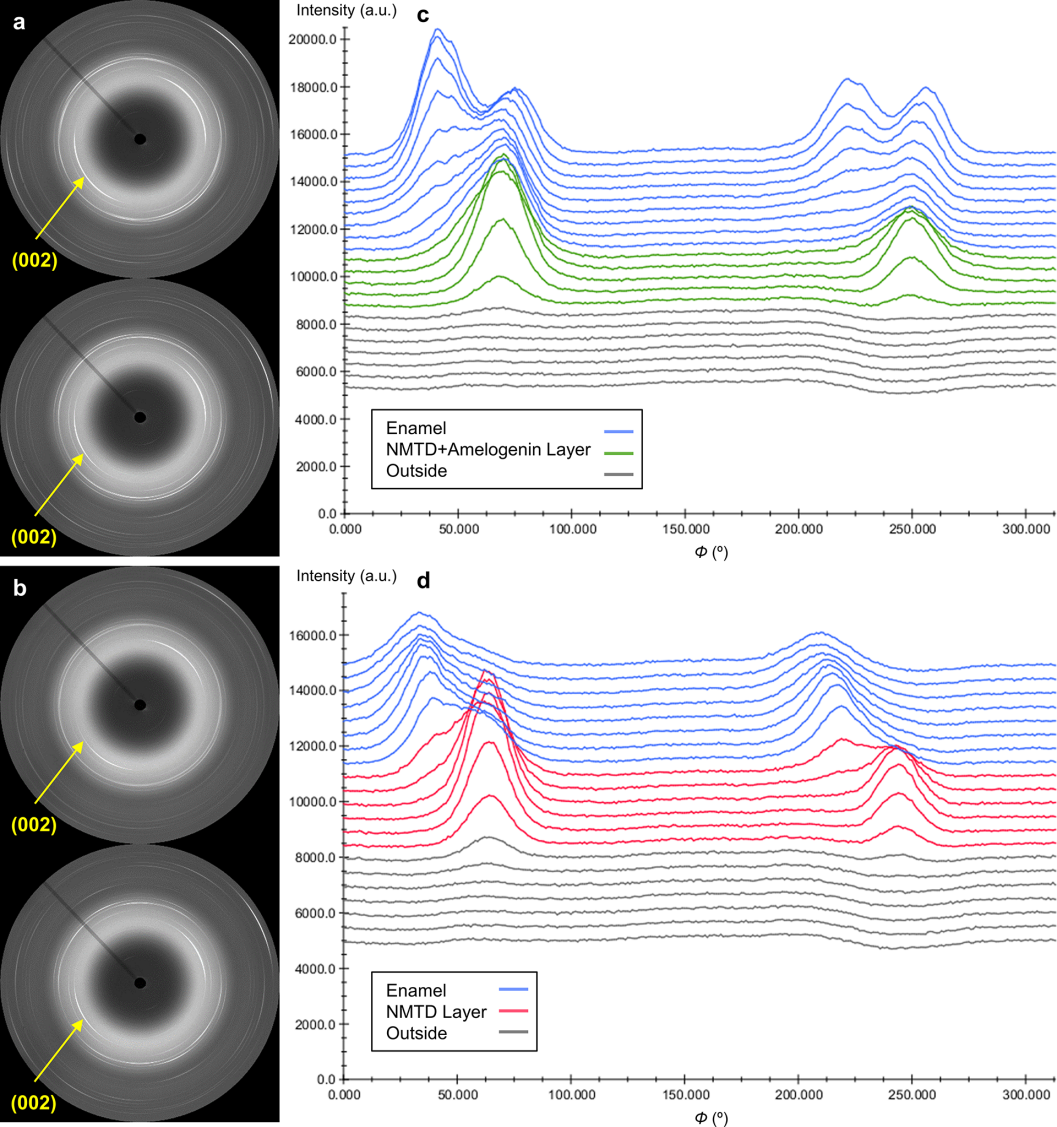
2D μXRD images from (a) a sample with amelogenin and (b) a sample without amelogenin (enamel point on the top and layer point on the bottom). Representation of the azimuthal plots from a line of (c) a sample with amelogenin and (d) a sample without amelogenin.

A high degree of crystalline anisotropy, as in dental enamel, produces a change in the intensity around the Debye ring of Bragg reflections in two dimensions that correlates with the degree of crystallite alignment or ordering.^53^ The intensity variations around the diffraction rings are indicative of tooth enamel texture.^51^ Diffraction spots in Figure 4a,b are concentrated in distinct arcs, both in the enamel and in the layers, which means that the crystals are ordered.^54^ The strongest texture (the most extreme intensity variation) for both kinds of samples (NMTD treatment with or without amelogenin) was found in the reflection (002), as can be appreciated in Figure 4a,b. The lattice plane reflection (002) does not overlap with other reflections and has the greatest intensity variation with maxima normal to the *c*-axis.^52^ Moreover, in the diffractograms shown in Figure 3 a shift was not observed in the (002) peak, since the contribution is due to the *c*-axis in this reflection and the *c*-axis dimension does not change from HA to FA.^3^ To analyze the different behavior of crystal orientation along the lines of points that enter into the teeth, the evolution of the azimuthal intensity of the reflection (002) has been plotted. This gives a linear representation of the evolution of the pixel intensity along the ellipse specified by the angle 2θ (Debye ring).^55^Figure 4c,d shows a typical example of the azimuthal plot for all the points of one line from both samples where there are two pronounced peaks separated by approximately 180°. The two pronounced peaks represent the opposing (002) reflection maxima that can be observed in the 2D μXRD images of Figure 4a,b. Sharp intense peaks are evidence of a strong preferred orientation, while broad peaks would indicate a more random orientation of the crystallites.^56^ It can be seen in Figure 4c,d that both kinds of layers have a strong preferred orientation with intense peaks, even exceeding the intensity of the underlying enamel. Moreover, the treatment with NMTD and amelogenin (Figure 4a,c) produces a layer that follows the preferred orientation of the surface enamel better than the treatment with only NMTD (Figure 4b,d), since the positions of the arcs and the peaks for the reflection (002) match better with enamel ones. The presence of another population of crystallites with distinct preferred orientation can be appreciated deeper in the enamel of the sample with protein where there are two additional peaks, also separated by approximately 180° (Figure 4c). This effect of four peaks due to two crystal populations with different preferred orientation is due to the natural structure of the enamel and can also be seen in the azimuthal plots of the enamel of both sample types in the Supporting Information Figure S3a,b.

**Figure 5:**
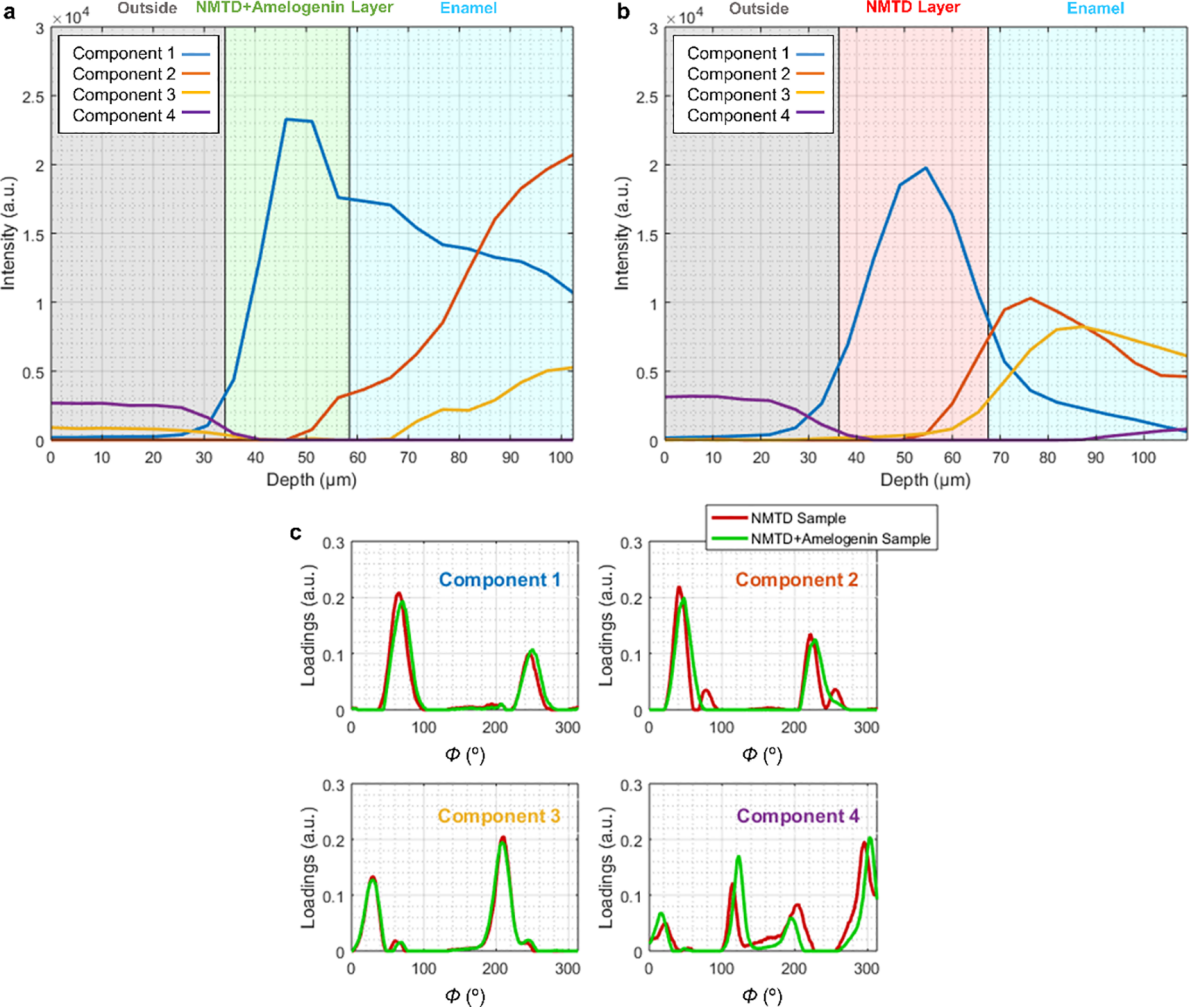
Examples of MCR analysis of the azimuthal plots from a line of (a) the sample with amelogenin and (b) the sample without amelogenin. (c) Loadings for each component of both samples, with amelogenin and without amelogenin.

In Figure 5, MCR analysis of the azimuthal plots of the reflection (002) from both treatments (NMTD with amelogenin and NMTD alone) is shown. MCR was developed by imposing non-negativity in both the intensities and azimuthal profiles. The orientation of component 1 (blue) is mostly present in the layer, while component 2 (orange) appears in the outer enamel and component 3 (yellow) becomes more important in the deeper enamel where new preferred orientations appear. Component 4 (purple) belongs to the points outside before reaching the samples. The evolution of the different components confirms that the orientation of the layer with NMTD and amelogenin (Figure 5a) follows the enamel underneath better than the layer with only NMTD (Figure 5b). In the NMTD layer with amelogenin, the change of the components 1 and 2 between the layer and the enamel is more gradual than in the one with NMTD alone, which is consistent with the movement of the peaks in Figure 4c,d. The changes in the intensities of the components as they enter deeper into the tooth (Figure 5a,b) can also be correlated with the intensities of the peaks in the azimuthal plots at different depths. The intensity in the layers of the azimuthal plots in Figure 4c,d is also higher than in the enamel. More examples are included in the Supporting Information Figure S3.

The loadings in Figure 5c show peaks separated by 180°, as the peaks of the original azimuthal plots. The peaks for components 1 and 2 are in similar positions, yet component 3 has them shifted to lower angles. This is consistent with component 3 being predominant in the inner enamel (Figure 5a,b) where azimuthal peaks appear at lower angles (Figure 4c,d).

## Conclusions

The combination of synchrotron infrared microspectroscopy and micro X-ray diffraction have shown to conform a useful methodology, together with the proper data treatment, to analyze the structure of apatites in samples of hard dental tissues. Infrared microspectroscopy provides information on chemical structural properties such as carbonate substitution, while micro X-ray diffraction allows to study the crystal structure and texture distribution on dental specimens. Synchrotron radiation allows one to detect small changes with better resolution and to analyze microzones with high signal intensity.

Since mature tooth enamel does not regenerate after substantial loss, finding a suitable solution for the dental problems is essential.^23,24,56^ The NMTD resin with or without amelogenin protein creates a remineralized layer on the surface of acid-etched teeth composed of carbonate free FA that has higher physicochemical stability than tooth HA. Nevertheless, amelogenin is a crucial component of this remineralizing product since it plays a critical role in controlling the preferred orientation of the growing crystal to resemble dental enamel. Further studies would be desirable to assess the clinical applicability of this biomimetic treatment in humans.

## Supporting Information Available

Experimental information; images of dental specimens prepared for the synchrotron techniques; second derivative spectra of the enamel and the different layers; and additional examples of azimuthal plots and MCR analysis for the different samples (PDF)
