# Supplementary material for: Combination of Two Synchrotron Radiation-Based Techniques and Chemometrics to Study an Enhanced Natural Remineralization of Enamel
Source: Anal Chem. 2022 Mar 23;94(13):5359–66. doi: 10.1021/acs.analchem.1c05498 (PMC8988122; doi:10.1021/acs.analchem.1c05498)
Supplement: Supplementary file 1 — ac1c05498_si_001.pdf [file ac1c05498_si_001.pdf]

SUPPORTING INFORMATION

Combination of Two Synchrotron Radiation-Based Techniques and Chemometrics to Study an Enhanced Natural Remineralization of Enamel

Sandra Diez-García[1(¥)], María-Jesús Sánchez-Martín[1*(¥)], José Manuel Amigo[2,3] and Manuel Valiente[1]

[1]GTS Research Group, Department of Chemistry, Faculty of Science, *Universitat Autònoma de Barcelona*, 08193 Bellaterra, Spain.

[2]Ikerbasque, Basque Foundation for Science, María Díaz de Haro, 48013 Bilbao, Spain

[3]Department of Analytical Chemistry, University of the Basque Country UPV/EHU, P.O. Box 644, 15 48080 Bilbao, Basque Country, Spain

(¥) Shared co-first authorship

* Co-first author and author for correspondence e-mail address: mariajesus.sanchez@uab.cat

Table of Contents:

1. Experimental Section S-2

1.1. Reagents S-2

1.2. Fluorapatite Reference Synthesis S-2

1.3. Amelogenin Protein Synthesis S-2

1.4. Specular Reflectance SR-µFTIR Experiment S-2

1.4.1. Data Acquisition S-2

1.4.2. Data Treatment S-3

1.5. Synchrotron tts-µXRD Experiment S-3

1.5.1. Data Acquisition S-3

1.5.2. Data Treatment S-3

2. References S-4

3. Figure S1. Images of dental specimens prepared for the synchrotron techniques S-5

4. Figure S2. Second derivative spectra of the enamel and the different layers S-5

5. Figure S3. Additional examples of azimuthal plots and MCR analysis for the different samples S-6

1. EXPERIMENTAL SECTION

1.1. Reagents

HEPES [4-(2-hydroxyethyl)-1-piperazine-ethanesulfonic acid] (99.5%), magnesium chloride hexahydrate (99%), calcium fluoride (95%) and chloramine T trihydrate (98-103%) were purchased from Sigma Aldrich (Steinheim, Germany); potassium dihydrogen phosphate (99.5%), calcium chloride dihydrate (74-78%) and tricalcium phosphate (35-40% (Ca)) from Panreac (Barcelona, Spain); potassium chloride (99-100.5%) from J. T. Baker (Deventer, Holland), all in powder form. Potassium hydroxide pellets (85%) and hydrochloric acid (37%) were purchased from Panreac (Barcelona, Spain). Deionized water was purified using a Millipore purification system (Millipore, Milford, MA, USA). The hydroxyapatite reference powder was of analytical grade and was used as received without further purification (>90%, Fluka, Sigma-Aldrich, Steinheim, Germany).

Food grade ion-exchange resins charged with different ions (Zn^{2+}, Ca^{2+}, F^- and PO_4^{3-}) were purchased from MionTec (Leverkusen, Germany). The base of the weak acid ion-exchange resins is a copolymer from acrylic acid, divinylbenzene and aliphatic diene with carboxylic acid functional groups (Lewatit S 8528, Lanxess, Leverkusen, Germany). While the base of the weak base ion-exchange resins is a styrene-divinylbenzene-copolymer with tertiary amine functional groups (Lewatit S 4528, Lanxess, Leverkusen, Germany). The different resins, ground to a particle size below 50 µm, were mixed to form the NMTD product, with a molar ratio between Ca^{2+}, F^- and PO_4^{3-} of 2:1:1, respectively. In addition, resin charged with Zn^{2+} ions is added, representing 0.2% of the dry weight of the resulting product.

1.2. Fluorapatite Reference Synthesis

The fluorapatite sample was synthetized by a solid phase reaction[1] mixing calcium fluoride (95%) and tricalcium phosphate (35-40% (Ca)) in the agate miller at the ratio of 1.67 Ca/P. Afterwards, the reagents were placed in the muffle furnace Selecta 366 PE (Selecta, Barcelona, Spain) to heat them at 1200 ^{o}C for 2 hours. The solid fluorapatite was then ground to powder for 15 minutes.

1.3. Amelogenin Protein Synthesis

A human 175 amino acid amelogenin (Swissprot Q99217, isoform 1, excluding the signal peptide) was expressed in *Escherichia coli* strain BL21 (DE3) and purified by an acid/heat treatment as described previously Svensson Bonde and Bulow[2]. Proteins were analyzed by matrix-assisted laser desorption/ionization, sodium dodecyl sulfate polyacrylamide gel electrophoresis and western blot to confirm the amelogenin production. Finally, recombinant amelogenin was quantified with a nanodrop.

Protein purification has been performed by the ICTS "NANBIOSIS", more specifically by the Protein Production Platform of CIBER in Bioengineering, Biomaterials & Nanomedicine (CIBER-BBN)/ IBB, at the UAB SePBioEs scientific-technical service (http://www.nanbiosis.es/portfolio/u1-protein-production-platform-ppp/).

1.4. Specular Reflectance SR-µFTIR Experiment

1.4.1. Data Acquisition

Specular reflectance spectra of the tooth samples were acquired using a Hyperion 3000 microscope coupled to a Vertex 70 spectrometer (Bruker, Ettlingen, Germany) and equipped with a Mercury-Cadmium-Telluride (MCT) detector. The microscope uses a 36x Schwarzschild objective (NA=0.52) coupled to a 36x Schwarzschild condenser to focus the synchrotron IR light

on the sample. Spectra were collected with OPUS 7.5 software (Bruker, Ettlingen, Germany) in the 700-4000 cm^{-1} range, at a spectral resolution of 4 cm^{-1}, with a masking aperture size of 6x6 μm^2, taking 256 co-added scans per spectrum to achieve a good signal-to-noise ratio and using a step size of 5x5 μm^2. A gold mirror was used as the reference to collect the background.

1.4.2. Data Treatment

OriginPro 8.5 software (OriginLab, Northampton, MA, USA) was employed to assign peak spectra numbers and perform maximum normalizations when comparing spectra. PCA was performed on these data on the mean centered spectra using PLS_Toolbox (Eigenvector Research, Wenatchee, WA, USA) working under MATLAB (The MathWorks, Natick, MA, USA).

1.5. Synchrotron tts-µXRD Experiment

1.5.1. Data Acquisition

The MSPD beamline is equipped with Kirkpatrick-Baez mirrors, providing a monochromatic focused beam of 15x15 μm^2 size at full width at half maximum and with a Rayonix SX165 CCD detector (round active area of 165 mm diameter, frame size 2048x2048 pixels, 79 mm pixel size and dynamic range 16 bit). The tooth sections were measured in transmission mode through the glass substrate.[3] The energy employed was 29.2 keV (λ=0.4246 Å), as determined from the Sn absorption K-edge. Instrumental calibration was carried out with a LaB_6 standard (NIST SRM 660b). The final data consisted of a two dimensions (2D) diffraction pattern in a series of points from outside to inside the tooth in lines separated by 200 µm. Reference compounds (hydroxyapatite and fluorapatite) were measured using a Kapton polyimide film as support material.

1.5.2. Data Treatment

The diffraction data of the samples were processed with the programs d1Dplot and d2Dplot[4], including the azimuthal plots, which consist of the evolution of pixel intensity along an ellipse specified by the Bragg angle 2θ and a given tolerance. This intensity was integrated over 360° in a narrow band containing the reflection with Miller indices (002) and then plotted versus the azimuthal angle Φ. The azimuthal plots of the reflection (002) were selected for this analysis since this reflection is normal to the *c*-axis of enamel crystallites.[5,6] MCR was performed using the same toolbox as before to study the evolution of the different orientations along the azimuthal plots of the reflection (002) collected from each line measured from the surface to the inside of the samples and with baseline correction. OriginPro 8.5 software was used to perform baseline correction and maximum normalization to the diffractograms when observing peak shifts.

2. References

(1) Wei, M.; Evans, J. H.; Bostrom, T.; Grondahl, L. Synthesis and Characterization of Hydroxyapatite, Fluoride-Substituted Hydroxyapatite and Fluorapatite. *J. Mater. Sci. Mater. Med.* **2003**, *14*, 311–320.

(2) Svensson Bonde, J.; Bulow, L. One-Step Purification of Recombinant Human Amelogenin and Use of Amelogenin as a Fusion Partner. *PLoS One* **2012**, *7* (3), e33269.

(3) Rius, J.; Vallcorba, O.; Frontera, C.; Peral, I.; Crespi, A.; Miravitlles, C. Application of Synchrotron Through-the-Substrate Microdiffraction to Crystals in Polished Thin Sections. *IUCrJ* **2015**, *2*, 452–463.

(4) Vallcorba, O.; Rius, J. D2Dplot: 2D X-Ray Diffraction Data Processing and Analysis for Through-the-Substrate Microdiffraction. *J. Appl. Crystallogr.* **2019**, *52* (2), 478–484.

(5) Al-Jawad, M.; Addison, O.; Khan, M. A.; James, A.; Hendriksz, C. J. Disruption of Enamel Crystal Formation Quantified by Synchrotron Microdiffraction. *J. Dent.* **2012**, *40* (12), 1074–1080.

(6) Siddiqui, S.; Anderson, P.; Al-Jawad, M. Recovery of Crystallographic Texture in Remineralized Dental Enamel. *PLoS One* **2014**, *9* (10), 1–9.

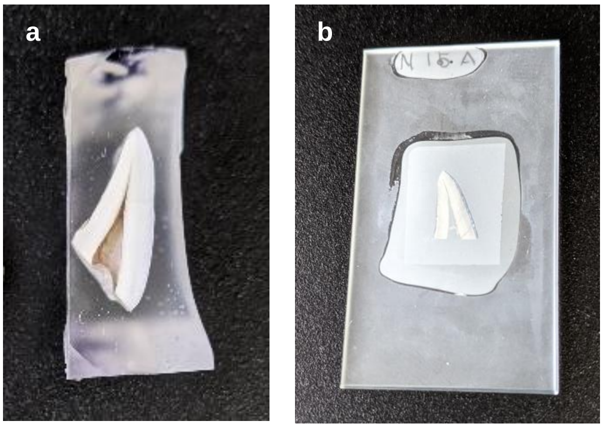

Figure S1. Image of a dental specimen prepared for (a) specular reflectance synchrotron radiation-based Fourier transform infrared microspectroscopy and (b) synchrotron through-the-substrate micro X-ray diffraction.

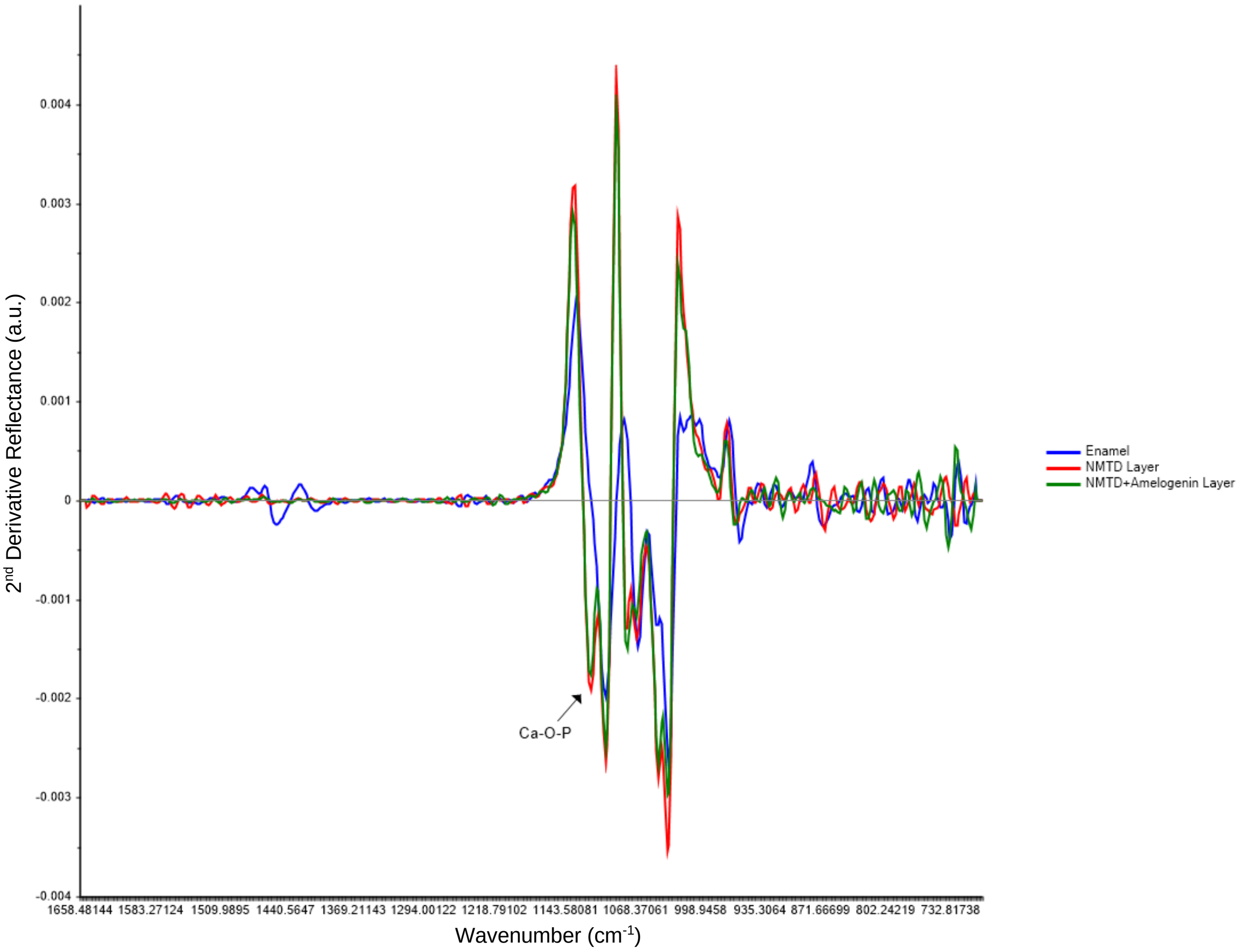

Figure S2. Savitzky-Golay second derivative average spectra of the enamel and the different layers performed with a polynomial order 3 on the region between 1660 and 700 cm^{-1} with Unscrambler X 10.4 software (n=11).

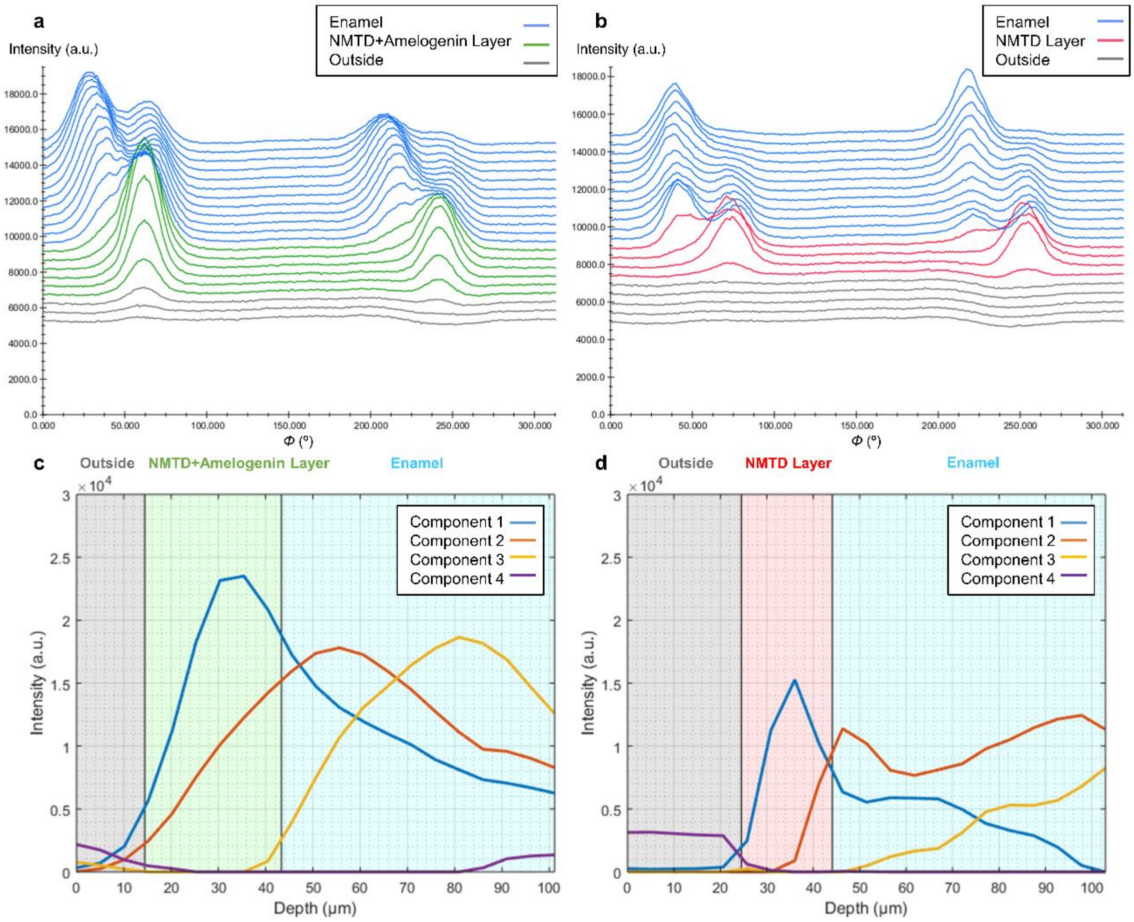

Figure S3. Representation of the azimuthal plots from a line of (a) a sample with amelogenin and (b) a sample without amelogenin. Examples of MCR analysis of the azimuthal plots from a line of (c) the sample with amelogenin and (d) the sample without amelogenin.
